# Supplementary material for: Upregulated CBX8 Promotes Cancer Metastasis via the WNK2/MMP2 Pathway
Source: Mol Ther Oncolytics. 2020 Oct 4;19:188–96. doi: 10.1016/j.omto.2020.09.012 (PMC7666318; doi:10.1016/j.omto.2020.09.012)
Supplement: Document S1. Table S1 and Figures S1–S4 [file mmc1.pdf]

OMTO, Volume *19*

Supplemental Information

Upregulated *CBX8* Promotes Cancer Metastasis via the *WNK2*/*MMP2* Pathway

Yongsheng Jia, Yujun Wang, Cuicui Zhang, and Mike Yue Chen

Table S1: Primers for CHIP

primers	Forward primer chain	Reverse primer chain
Primer 1	CACCTCACACCTGTTAAGATC	CACTCACACCACACTGCAC
Primer 2	GCCAAGATATGGAAACAACC	GTACTCCACTGTGCAAAC
Primer 3	CCAGGCACAGAAAGACTAC	CAACTTCCATTCTGCTCTCTGC
Primer 4	GTGTTAAGACAGCAGATCTC	CCTAGGGCTGTGTGATGGCGTC
Primer 5	CACGGGCCTGCACGCACTGTG	CATTCTGTGTTGGTACTCAC
Primer 6	TGGGCAACATGGTGAAA	AAGCAATCCTCCCACCTCA
Primer 7	CAGAGGTTGCAGTGAGGCGAG	GATGGTCTGGCTCTGTCAC
Primer 8	GTCCCTGTGAGCCTGGTGGAG	CCTGTGGGAGGAATCCAGAACC
Primer 9	GAGCTCCTGCACAAGGCACTGG	AGGAGCCTGAAGCCTTCAGACC
Primer 10	GCCACAGCTGATGGTCACACG	GAGCCACCAGCTTCGCAACTCC
Primer 11	GAGAAGACCAGCACGCTGG	GTACCAGGGTACTCCAGTCC

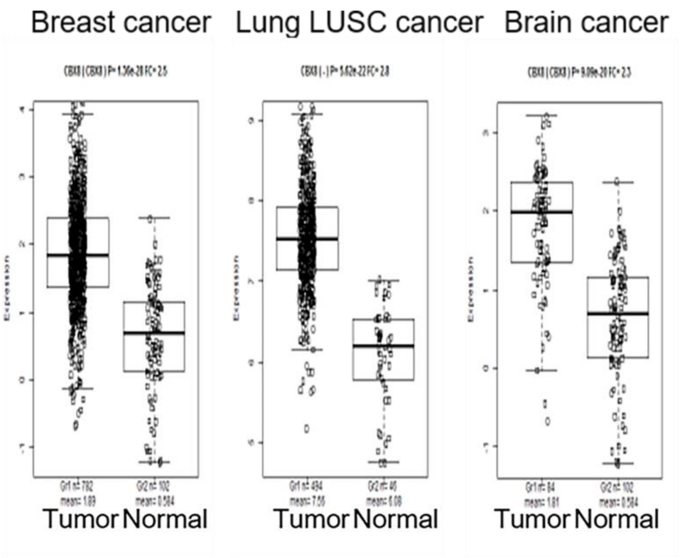

Figure S1. *CBX8* expression is analyzed in breast cancer, lung LUSC cancer, and brain cancer tissues compared to normal tissues. *CBX8* shows significant upregulation from our in-house ANOVA difference gene expression analysis using TCGA GDAC provisional patient cohorts for brain cancer (GBM with 525 tumor vs 10 normal samples), breast cancer (BRCA, with 1100 tumor vs 112 normal samples), and lung cancer (LUAD, with 517 tumor vs 59 normal samples).

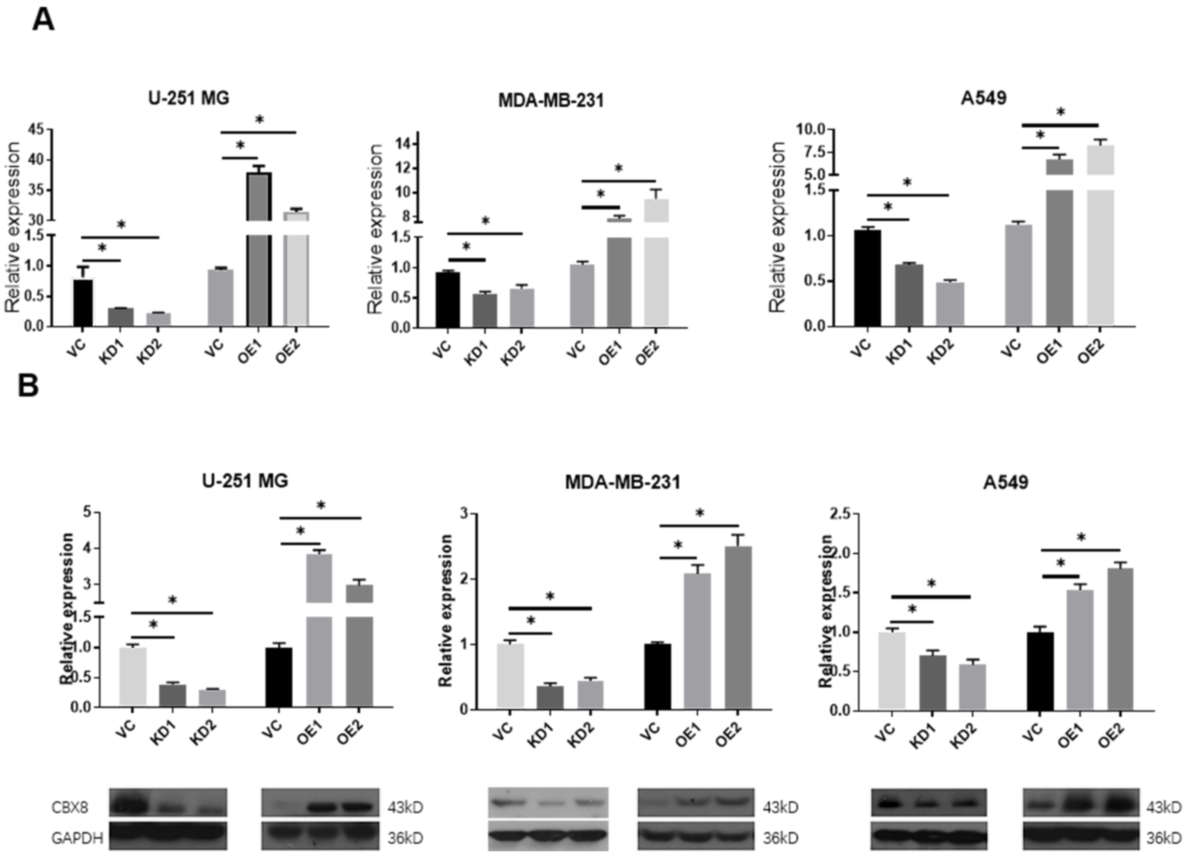

Figure s2: *CBX8* modulated in cancer cells and evaluated by qRT-PCR (A) and western blot (B). “*” indicated $p<0.05$.

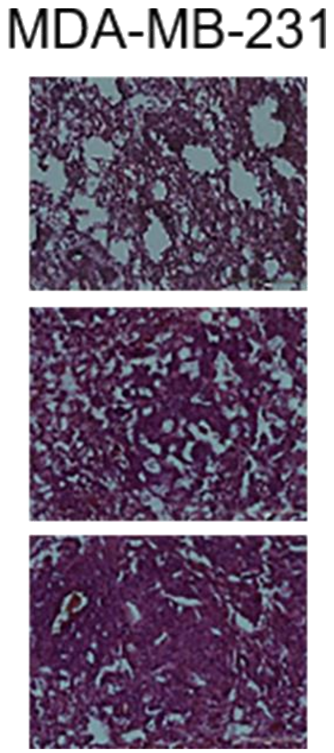

Figure S3. H&E staining of lung tissues from mice injected with CBX8-modulated MDA-MB-231 cells as well as corresponding vector controls.

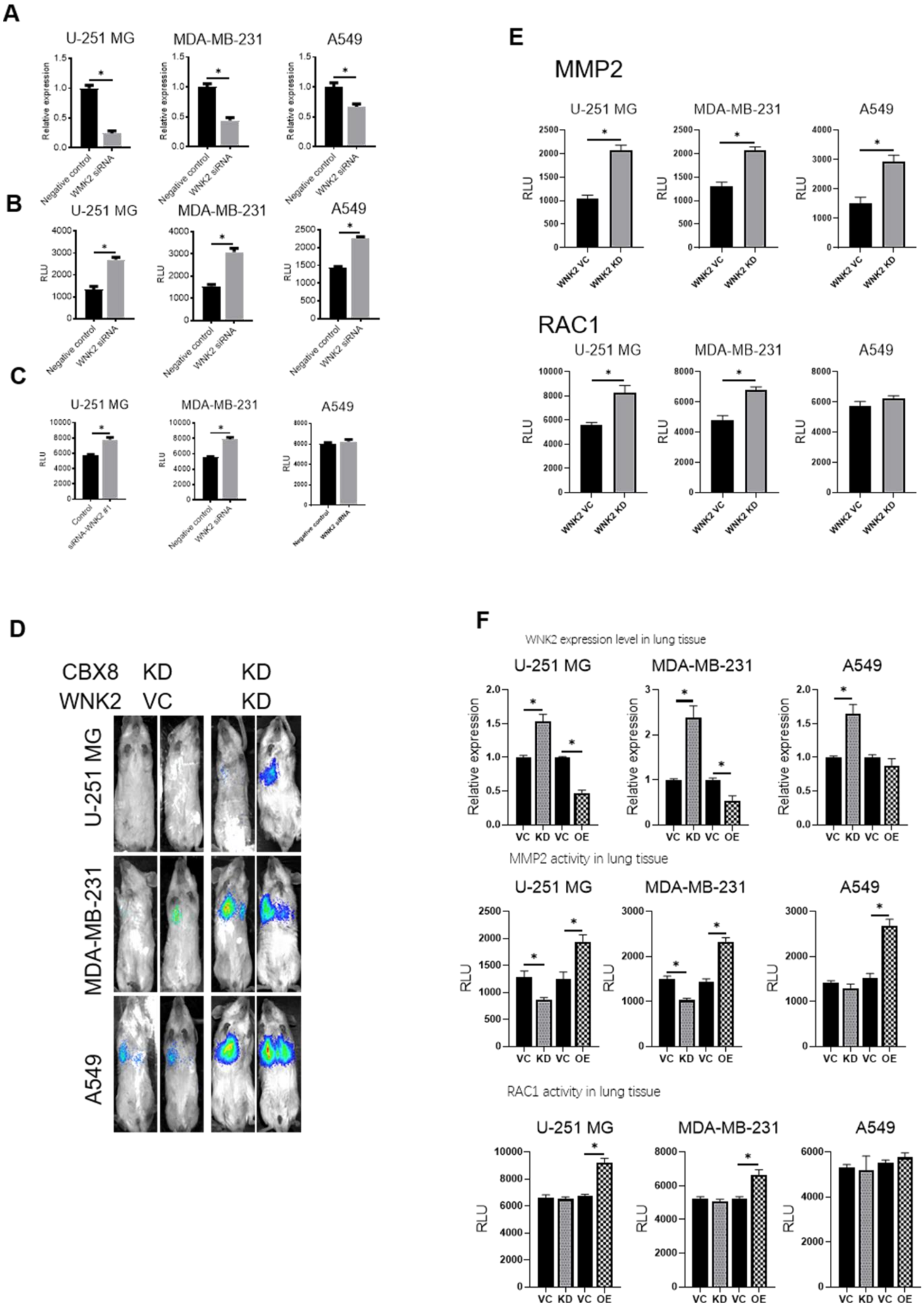
A
U-251 MG
MDA-MB-231
A549
Relative expression
Negative control
WMK2 siRNA
WNK2 siRNA
B
U-251 MG
MDA-MB-231
A549
RLU
Negative control
WNK2 siRNA
C
U-251 MG
MDA-MB-231
A549
RLU
Control
siRNA-WNK2 #1
Negative control
WNK2 siRNA
D
CBX8 KD KD
WNK2 VC KD
U-251 MG
MDA-MB-231
A549
E
MMP2
U-251 MG
MDA-MB-231
A549
RLU
WNK2 VC
WNK2 KD
RAC1
U-251 MG
MDA-MB-231
A549
RLU
WNK2 VC
WNK2 KD
F
WNK2 expression level in lung tissue
U-251 MG
MDA-MB-231
A549
Relative expression
VC KD VC OE
MMP2 activity in lung tissue
U-251 MG
MDA-MB-231
A549
RLU
VC KD VC OE
RAC1 activity in lung tissue
U-251 MG
MDA-MB-231
A549
RLU
VC KD VC OE

Figure S4. Knockdown of *WNK2* increases the RAC1 and MMP2 activity in tumor cells A. Transient transfection of SiRNA targeting *WNK2* suppress its expression. B and C. Knockdown of *WNK2* enhances the MMP2 and RAC1 activity in U-251 MG, MDA-MB-231, and A549 tumor cells. WNK2 promoter activity increased in U-251MG, MDA-MB-231 and A549 cells after knockdown of CBX8(E). The expression of WNK2, MMP2 and RAC1 are assessed in the tumor tissues of the in vivo mouse model(F). “*” indicated $p<0.05$.
