# Supplementary material for: Upregulated CBX8 Promotes Cancer Metastasis via the WNK2/MMP2 Pathway
Source: Mol Ther Oncolytics. 2020 Oct 4;19:188–96. doi: 10.1016/j.omto.2020.09.012 (PMC7666318; doi:10.1016/j.omto.2020.09.012)
Supplement: Document S2. Article plus Supplemental Information [file mmc2.pdf]

Molecular Therapy
Oncolytics
Original Article

Upregulated *CBX8* Promotes Cancer Metastasis via the *WNK2/MMP2* Pathway

Yongsheng Jia,[1,2,6] Yujun Wang,[2,6] Cuicui Zhang,[3,5,6] and Mike Yue Chen[2,4]

[1]Thyroid and Neck Department, Tianjin Medical University Cancer Institute and Hospital, National Clinical Research Center for Cancer, Tianjin, China; [2]Division of Neurosurgery, City of Hope and Beckman Research Institute, Duarte, CA, USA; [3]Department of Thoracic Oncology, Tianjin Medical University Cancer Institute and Hospital, Tianjin, China; [4]Key Laboratory of Cancer Prevention and Therapy, Tianjin, China; [5]Tianjin's Clinical Research Center for Cancer, Tianjin, China

Metastasis is associated with poor prognosis in cancer and is a multistep process that includes invasion and migration. Several epigenetic factors are involved in this process, including chromobox protein homolog 8 (*CBX8*). Here, we show that *CBX8* is overexpressed in many cancers compared with normal tissues. Functional analyses indicated that *CBX8* promoted invasion and migration in glioblastoma, breast cancer, and lung cancer *in vitro* and *in vivo*. *WNK2* was identified as a target gene of *CBX8*, which interacted with the *WNK2* promoter to suppress *WNK2* expression and activity. *WNK2* acted as an antioncogene, and decreased *WNK2* levels resulted in high activity of matrix metalloprotease (MMP)-2 and *RAC1*, which play a central role in invasion and migration, respectively. There was a positive relationship between *MMP2* and *RAC1* activity in *CBX8*-modulated cell lines. In addition, *WNK2* negatively regulated *MMP2* and *RAC1* activity. Collectively, the results indicated that *CBX8* promoted invasion and migration by targeting *WNK2*, which resulted in increased *RAC1* and *MMP2* expression and activity. Therefore, *CBX8* may be a novel therapeutic target to treat metastatic cancers.

INTRODUCTION

Metastasis is associated with advanced tumors and is the main cause of cancer-related death. It occurs in all malignant tumors and in certain benign diseases, such as endometriosis and tuberous sclerosis complex.[1,2] It is an integrated process that involves several biological mechanisms, such as digestion of the basement membrane and extracellular matrix (ECM), separation from the primary tumor, penetration into a blood vessel through transendothelial migration, and proliferation at distant organs.[3,4] Invasion and migration play a crucial role in this multistep process and are central features of cancer cells. They can contribute to metastasis individually or in combination.[5,6] For instance, invasion triggers metastasis by providing space and direction for cancer cell migration by digesting the basement membrane and the ECM. Since they are oriented to a blood vessel or lymph vessel, cancer cells can penetrate them by changing their morphology.[5] Since invasion and migration function simultaneously at critical steps of metastasis, they should be investigated together to obtain further insight.

Many genes and pathways are involved in invasion, migration, and metastasis; specifically, matrix metalloprotease 2 (*MMP2*) and Ras-related C3 botulinum toxin substrate 1 (*RAC1*) play a pivotal role in invasion and migration.[7,8] *MMP2* is a member of the MMP family that functions in ECM degradation. *MMP2* can degrade the basement membrane by targeting its most abundant component, type IV collagen.[9] Similarly, *RAC1* plays an important role in migration.[10] It is a member of the *RAC* subfamily and promotes F-actin polymerization to reorganize the cytoskeleton, which functions in migration.[11,12] *MMP2* and *RAC1* activity is negatively regulated by *WNK2*, a member of the WNK (with no K = lysine) family of protein kinases.[13,14] Outside of conventional gene mutations, epigenetic mechanisms play an important role in the regulation of *WNK2* expression.[15]

Chromobox protein homolog 8 (*CBX8*), together with *CBX2*, *CBX4*, *CBX6*, and *CBX7*, forms the CBX family, which is the central part of the polycomb repressive complex 1 (PRC1).[16] PRC1 and PRC2 can epigenetically regulate genes by methylating histones, such as di- or tri-methylation of lysine 27 on histone H3 (H3K27me2/me3), which results in chromatin remodeling and silencing of genes.[17,18] However, *CBX8* can also regulate gene expression in a PRC1-independent manner.[19,20] Recent studies show that increased expression of *CBX8* is associated with many types of cancer, though these findings require additional characterization and functional analyses.[21–23] Here, we explored the functional mechanism of *CBX8* in cancer to determine its association with metastasis.

RESULTS

CBX8 Is Increased in Cancer

To explore the expression pattern of *CBX8* in cancer, we performed a literature search on The Cancer Genome Atlas database and compared *CBX8* expression levels between tumor tissues and normal tissues. The results showed that *CBX8* was expressed at low levels in most normal tissues except the testis, pituitary, and fibroblasts. However, *CBX8* was overexpressed in most cancers compared with corresponding normal tissues (Figure 1A). Considering the *CBX8*

Received 15 March 2020; accepted 30 September 2020;
https://doi.org/10.1016/j.omto.2020.09.012.

[6]These authors contributed equally to this work.

Correspondence: Mike Yue Chen, Division of Neurosurgery, City of Hope and Beckman Research Institute, City of Hope, Duarte, CA 91010.
E-mail: mikeyuechen@163.com

This is an open access article under the CC BY license (http://creativecommons.org/licenses/by/4.0/).

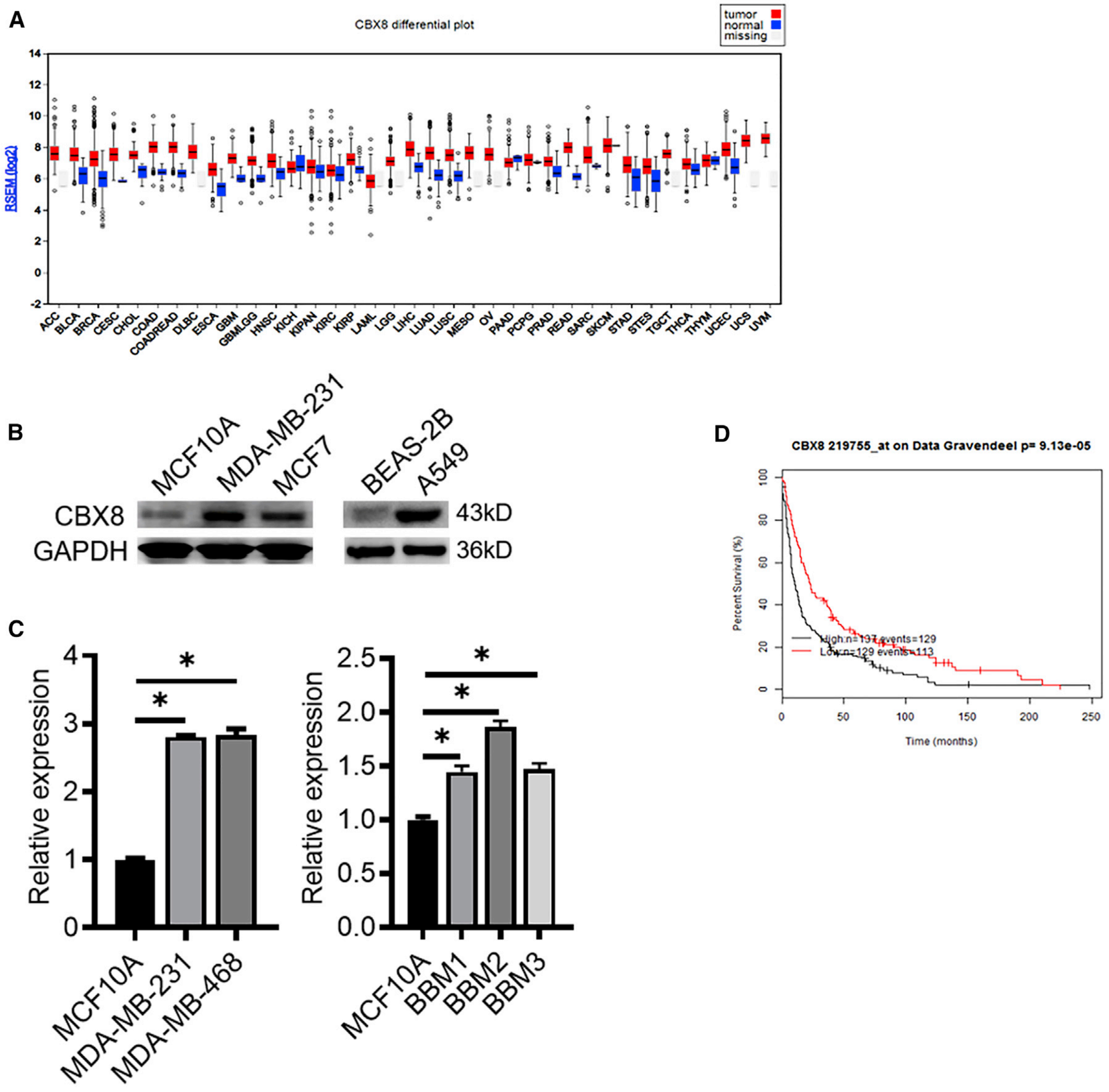

Figure 1. *CBX8* Expression Is Upregulated in a Variety of Cancers

(A) *CBX8* shows higher mRNA-seq expression profile comparing multiple human cancer tissues (in red color) versus normal tissues (in blue) as well as gray (missing TCGA normal sample type). The figure was generated from the COH Bioinformatics Core using Broad Institute TCGA Genome Data Analysis Center (2016): Analysis-ready standardized TCGA data from Broad GDAC Firehose 2016_01_28 run; Broad Institute of MIT and Harvard. Dataset (https://doi.org/10.7908/C11G0KM9). (B and C) Comparison of *CBX8* expression in breast cancer, lung cancer, and primary breast to brain metastatic cancer cells via (B) western blotting, and (C) qRT-PCR analysis. $^{*}p < 0.05$. (D) Kaplan-Meier overall survival analysis shows that glioma patients with higher *CBX8* expression (n = 131) displayed significantly poorer overall survival than those with lower *CBX8* expression (n = 135; p = 1.94e−7) using Glioma Affymetrix microarray cohorts.

expression profile and clinical implications, we further examined its expression in brain tumors (data published in Tang et al.[24]), breast cancer, and lung cancer. In each group, *CBX8* expression was significantly higher in tumor tissues than in normal tissues (Figure S1), and glioma patients with higher *CBX8* expression displayed significant poorer overall survival than those with lower *CBX8* expression (Figure 1D). *CBX8* expression was higher in breast cancer and lung cancer cell lines than in non-cancer cell lines (Figures 1B and 1C). Taken together, these data indicated that *CBX8* has a potential role in tumorigenesis.

CBX8 Promotes Migration and Invasion *In Vitro*

CBX8 knockdown and overexpressing cell lines were generated in glioblastoma (U-251 MG), breast cancer (MDA-MB-231), and lung cancer (A549), and *CBX8* expression levels were validated by quantitative reverse-transcriptase PCR (qRT-PCR) and western blotting

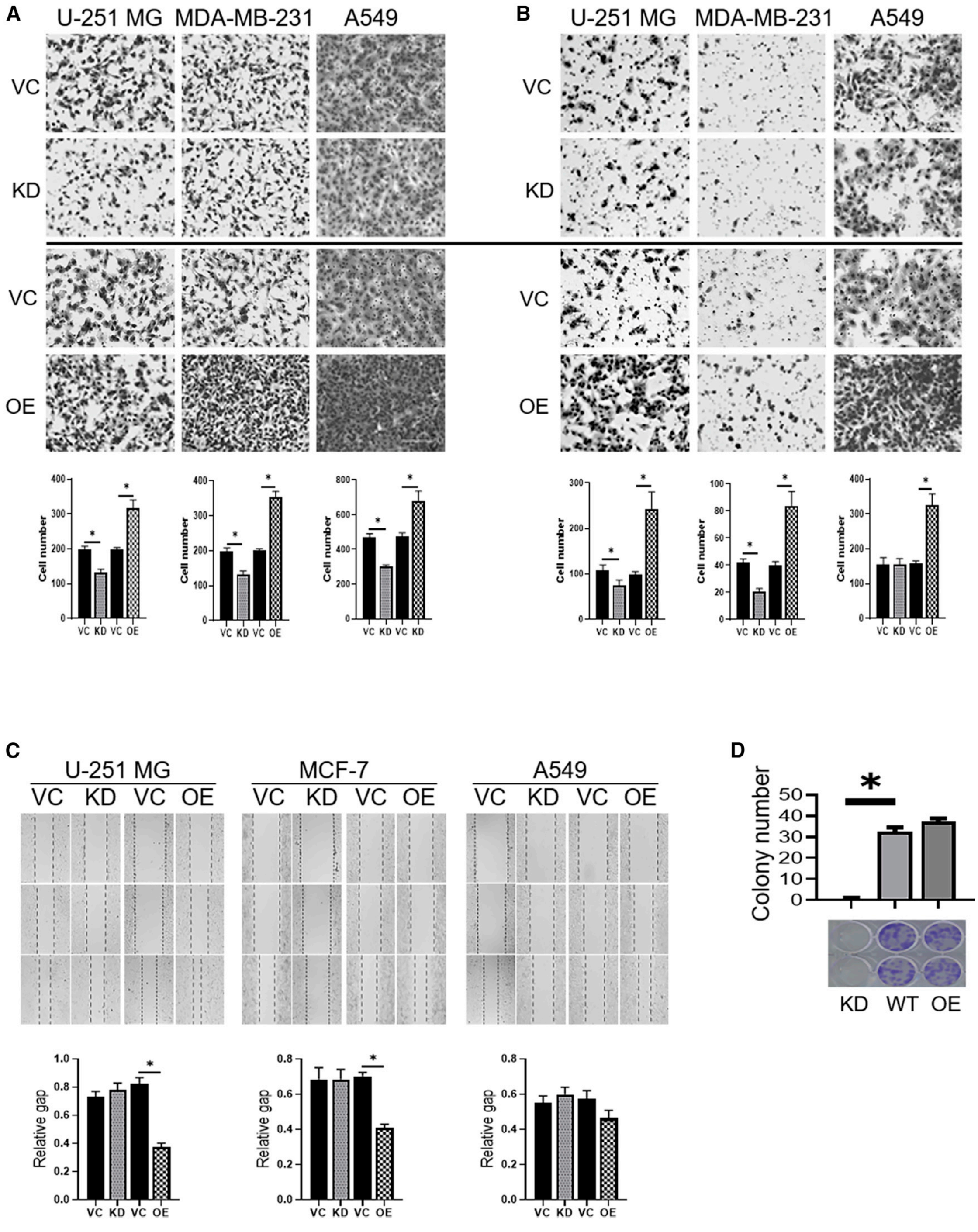

Figure 2. Effects of *CBX8* Modulation on the Invasion and Migration of U-251 MG, MDA231-MB, and A549 Cells via Transwell Assay

A total of 8,000 cells/well for migration and 50,000 cells/well for invasion in no serum medium were seeded in the upper chamber, respectively. After 8 h incubation for migration or 20 h for invasion, cells passing through the lower chamber membrane were counted, and corresponding images (10×) were captured in at least three random fields. Representative images and quantification of cell numbers are presented here. Overexpression (OE) of *CBX8* significantly increased migration and invasion capacity of U-251 MG, MDA231-MB, and A549 cells, whereas silencing (knockdown [KD]) *CBX8* decreased migration and invasion. (A) Transwell migration assay. (B) Transwell invasion assay. *$p < 0.05$. (C) Scratch assay images of U-251 MG, MCF-7, and A549 with *CBX8* modulation (KD, OE) were analyzed based on the online platform WimScratch compared to corresponding wild-type (WT) cells. (D) Effect of *CBX8* modulation on proliferative capacity of MDA-MB-231 cells with *CBX8* silencing (KD) or OE. Images of representative wells from colony-formation assay and corresponding graph are shown. Colonies were stained with 0.1% crystal violet. Data are representative of three independent experiments performed in triplicate. *$p < 0.05$.

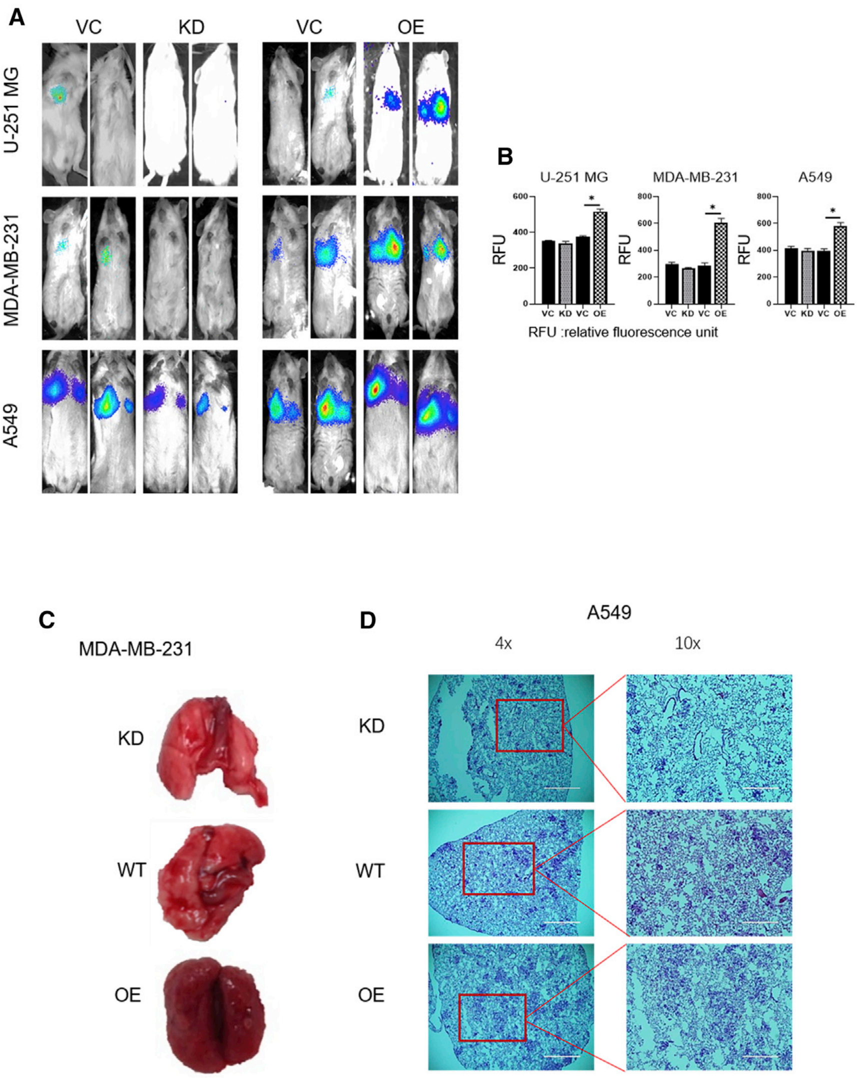

Figure 3. Effects of *CBX8* Modulation on Tumor Metastasis via Tail Vein Model

(A and B) Representative bioluminescent images (A) and bioluminescent density (B) of mice (n = 8) injected with *CBX8*-modulated (KD, OE) U-251 MG, MDA-MB-231, and A549 cells as well as corresponding WT controls. *$p < 0.05$. (C) Representative images of lung tissues of NSG mice injected with *CBX8*-modulating MDA-MD-231 cells. (D) H&E staining of lung tissues from mice injected with *CBX8*-modulated MDA-MB-231 and A549 cells as well as corresponding vector controls.

(Figure S2). Further, the Transwell invasion assay was performed in *CBX8*-modulated U-251 MG, MDA-MB-231, and A549 cell lines. In U-251 MG and MDA-MB-231 cells, invasion was significantly higher in the *CBX8* overexpression group than in the control and silencing groups ($p < 0.05$). An increasing trend of invasion was found between the control group and the *CBX8* silencing group, although this was not statistically significant. In A549 cells, invasion increased with increasing *CBX8* expression, although the difference was not statistically significant. This may be attributed to the high standard deviation (Figure 2B). Consistent with the results of the Transwell invasion assay, *CBX8* expression was positively associated with migration, as shown in the Transwell migration assay (Figure 2A) and scratch assay (Figures 2C and S3). Statistically significant differences were observed in the overexpression group versus the control group and the overexpression group versus the silencing group in the three cell lines in the migration assay. The scratch assay was performed to measure the mobility of the cells after modulation of *CBX8* expression. In U-251 MG and MDA-MB-231 cells, the mobility was significantly increased in the overexpression group compared with the control and silencing groups. In A549 cells, an increasing trend in mobility was observed in correlation with increasing *CBX8*. The results thus showed that *CBX8* expression was positively associated with invasion and migration. The effect of *CBX8* modulation on proliferative capacity of MDA-MB-231 cells was evaluated with colony-formation assay and indicated *CBX8* promoted proliferation (Figure 2D).

CBX8 Increases Metastasis *In Vivo*

We next generated an animal model to evaluate the functional role of *CBX8* in metastasis. The luciferase gene was transfected into *CBX8*-modulated cell lines (U-251 MG, MDA-MB-231, and A549) and injected into NOD scid gamma (NSG) mice through the tail vein. Luciferase imaging was performed 28–35 days after injection to monitor the location and growth of cells in the lung (Figures 3A and 3B). The results showed that luciferase count and density were increased in relation to *CBX8* upregulation, especially in the *CBX8* overexpression group. Hematoxylin and eosin (H&E) staining showed that both the tumor number and the area of the tumor locus were increased in association with *CBX8* upregulation (Figures 3C, 3D, and S3).

CBX8 Suppresses *WNK2* Directly and Increases *MMP2* and *RAC1*

Chromatin immunoprecipitation sequencing (ChIP-seq) data indicate that *CBX8* can bind to *WNK2*.[25] A systematic literature search showed that *WNK2* is a potential target of *CBX8*. To determine whether reduced *WNK2* expression was associated with increased *CBX8* expression, *CBX8* and *WNK2* expressions were analyzed in U-251 MG, MDA-MB-231, and A549 cells under *CBX8* modulation. The results showed that *WNK2* had a negative relationship with *CBX8*, indicating that *CBX8* may suppress *WNK2* (Figure 4A). To further address the association between *CBX8* and *WNK2*,

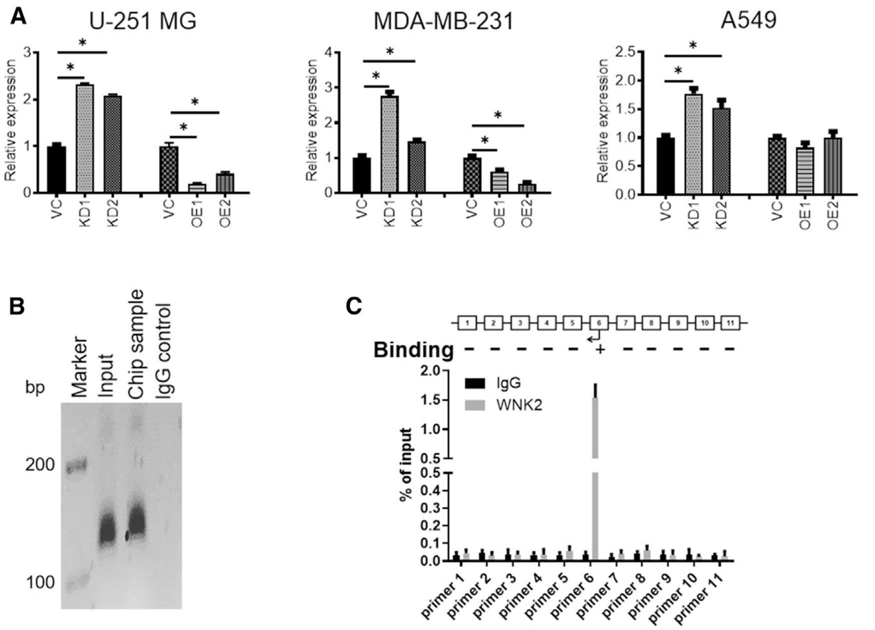

Figure 4. *WNK2* Is a Target of *CBX8*. (A) *WNK2* Expression Is Directly Impacted under *CBX8* Modulation in Different Tumor Types

(A) qRT-PCR indicates that *WNK2* is significantly upregulated in *CBX8* silencing (KD) tumor cells, while there is striking downregulation in U-251 MG and MDA-MB-231 cells with *CBX8* OE. *p < 0.05. (B) Gel images of PCR products and qRT-PCR graph. (C) From ChIP assay using *CBX8* OE U-251 MG cells. With the positive input control, *CBX8* promoter sequence located between primers was detected in 11 *CBX8* pull-down products compared to the negative IgG control.

ChIP was performed in *CBX8*-overexpressing cell lines (U-251 MG) (Figures 4B and 4C). The results indicated that *CBX8* can directly interact with the promoter of *WNK2*, which is consistent with the *CBX8* suppressing function. To examine the effect of *WNK2* on *MMP2* and *RAC1*, *WNK2* was knocked down and *MMP2* and *RAC1* activities were analyzed. The results confirmed that *WNK2* negatively regulated *MMP2* and *RAC1* (Figures S4A–S4C and S4E).

MMP2 and *RAC1* Are Upregulated in Association with Increasing *CBX8* Levels

To explore the involvement of *CBX8* in invasion and migration, we performed a literature search and tested potential genes associated with these processes. The results showed that *MMP2* and *RAC1* changed considerably in response to the modulation of *CBX8* expression. The *MMP2* expression pattern was evaluated by qRT-PCR in all *CBX8*-modulated cell lines. The results showed that *MMP2* expression and activity increased in association with *CBX8* upregulation (Figures 5A, 5B, and S4F). Similarly, *RAC1* activity increased in association with *CBX8* upregulation, though a mild change was observed in the *RAC1* expression pattern (Figure 5C, 5D, and S4F).

DISCUSSION

As the leading cause of death, metastasis has been extensively reported in the literature. However, the metastatic process is complex, and the underlying mechanism remains elusive. Invasion and migration are critical biological properties of cancer cells and integrated processes that are difficult to separate from each other. In addition to other properties such as colonization, the cooperation between invasion and migration is crucial to promote metastasis. Recently, epigenetic mechanisms, involving *CBX8*, were shown to play an important role in metastasis.[26,27]

CBX8 acts as an oncogene and is involved in tumor progression.[21–23] Alterations in *CBX8* are observed in many cancers, such as glioblastoma, colorectal cancer (CRC), breast cancer, leukemia, and hepatocellular carcinoma.[22–24,28,29] Specifically, *CBX8* promotes proliferation, differentiation, invasion, and metastasis, promoting breast tumorigenesis and increasing proliferation in CRC.[22,24] However, the role of *CBX8* in metastasis is context dependent. Yuan et al.[30] reported that *CBX8* promotes invasiveness in bladder cancer. However, in esophageal squamous cell carcinoma (ESCC) and CRC, *CBX8* suppresses metastasis by inhibiting Snail and p53.[24,31] Most studies found CBX8 acted as an oncogene in cancers. In one type of cancer, CBX8 may even have a function of promoting proliferation and inhibiting metastasis.[24,31] CBX8 has a function through various pathways in different cancers. However, the mechanism is not reported clearly in each type of cancer. Here, we explored the role of *CBX8* in glioblastoma, breast cancer, and lung cancer and found that the efficiency for promoting metastasis differs among cancers. The effect of *CBX8* on metastasis was more significant in glioblastoma and breast cancer than in lung cancer. Additionally, though sharing a positive relationship with both, *CBX8* had a stronger effect on invasion than migration.

CBX8 functions through PRC1-dependent and PRC1-independent pathways.[17–20] In the PRC1-dependent pathway, *CBX8* suppresses genes by assembling the PRC1 complex with other proteins. However, an increasing number of studies showed that *CBX8* can function in a PRC1-independent manner, such as *CBX8* binding to Snail in ESCC, to p53 in CRC, and to p16Ink4a in CRC.[20,31] Here, we showed that *CBX8* can suppress *WNK2* expression by binding to the promoter of *WNK2*.

WNK2 is a kinase that negatively regulates invasion and migration by suppressing *MMP2* and *RAC1* expression and activity.[13,14] However, the low activity of WNK2 in cancer is associated with low levels of expression rather than changes in gene sequence, indicating that an epigenetic mechanism is involved in the regulation of *WNK2*.[15] *CBX8* mutations are rarely reported, whereas overexpression of

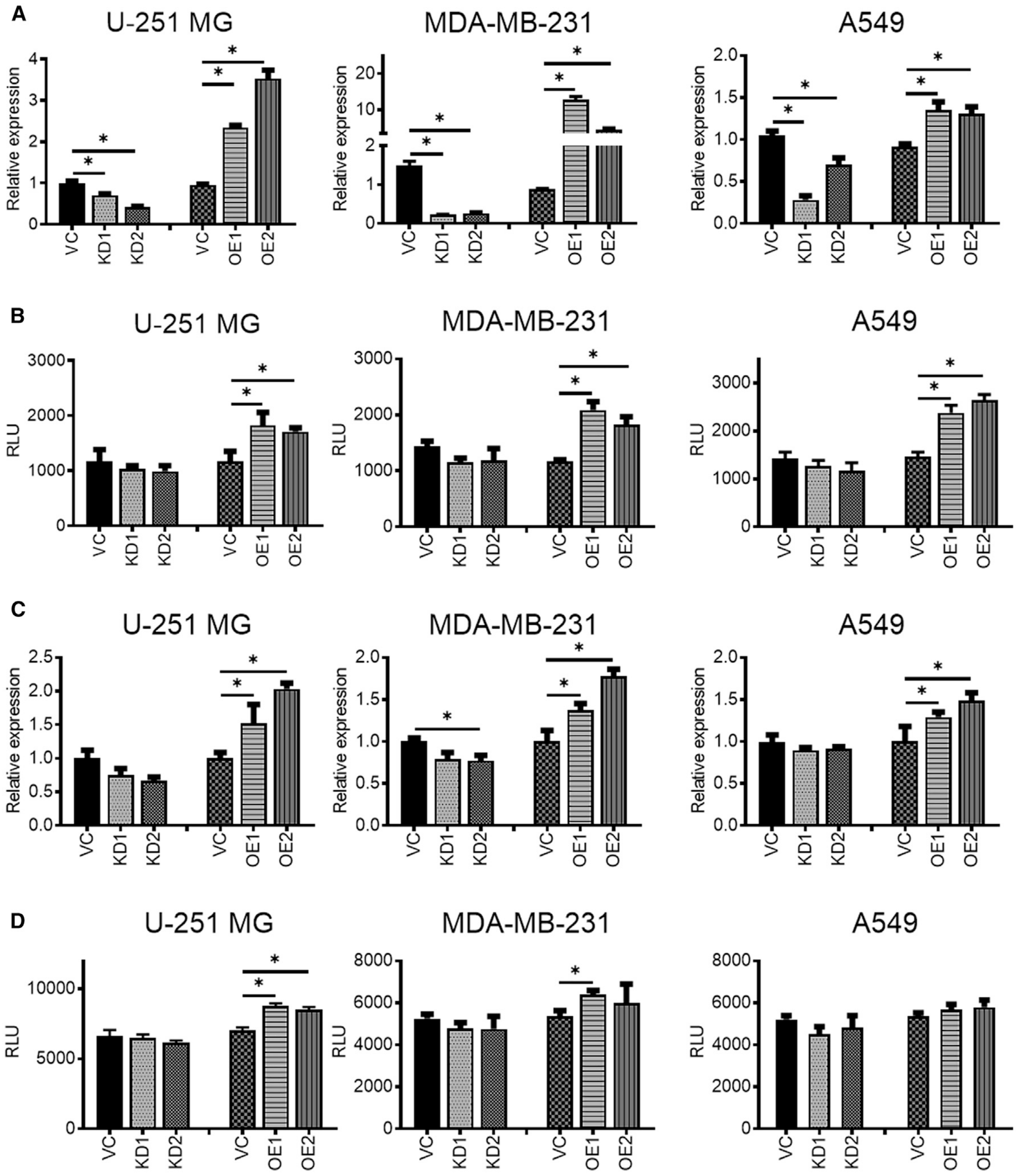

Figure 5. *CBX8* Modulation (KD, OE) Has a Positive Relation with the Expression and Activity of *MMP2* and *RAC1*

(A–D) qRT-PCR (A and C) and protein activity assay (B and D) suggest that increased *CBX8* induces the expression level and protein activity of MMP2 in U-251 MG, MDA-MB-231, and A549 cells, whereas knockdown of *CBX8* has a suppressive effect. $^{*}p < 0.05$.

CBX8 is widely found in various cancers.[22–24,28,29] Overexpressed *CBX8* may function in cancer by suppressing antioncogenes. Here, we showed that *CBX8* suppressed *WNK2* expression by binding to the *WNK2* promoter, increasing *MMP2* and *RAC1* expression and activity.

As a member of the MMP family, *MMP2* plays a central role in malignant tumors and predicts a poor prognosis. *MMP2* promotes invasion by promoting ECM degradation to provide space and direction for cell movement and digestion of the basement membrane to maintain tissue organization.[9] *RAC1* mainly regulates cancer cell

Table 1. Antibiotic Concentrations Used for Selection after Transfection

Cell Type	G418 (overexpressing)	Puromycin (silencing)
U251MG	1,000 μg/mL	2 μg/mL
MDA-MB-231	1,200 μg/mL	2 μg/mL
A549	1,000 μg/mL	2 μg/mL

migration. Both expression and activity of *MMP2* and *RAC1* are negatively regulated by *WNK2*.[13,14] After validating the negative relationship between *CBX8* and *WNK2*, we found that *MMP2* expression and activity were increased in association with *CBX8* overexpression. Both invasion and migration increased, indicating that *CBX8* can suppress *WNK2* and promote invasion and migration by increasing the expression and activity of *MMP2* and *RAC1*.

Collectively, the results indicated that *CBX8* was upregulated in glioblastoma, breast cancer, and lung cancer. Overexpression of *CBX8* can promote cancer cell invasion and migration, leading to metastasis. This process is dependent on the *CBX8*-mediated suppression of *WNK2*, which results in increased expression and activity of *MMP2* and *RAC1*.

MATERIALS AND METHODS

Cell Lines and Cell Culture

U-251 MG, MDA-MB-231, and A549 cells were purchased from American Type Culture Collection (ATCC, Manassas, VA, USA). U-251 MG and MDA-MB-231 cells were grown in Dulbecco's modified Eagle medium (DMEM); A549 cells were grown in F-12K Nutrient Mixture, Kaighn's Mod (FK-12). All media contained 10% fetal bovine serum (FBS) (Omega Scientific) and 1% penicillin-streptomycin. *CBX8*-overexpressing and -silencing plasmids were purchased from OriGene (overexpression: RC203417L1; knockdown: TF317361). Plasmids with a scramble sequence were used as the silencing group control, and those with a flag tag sequence were used as the overexpressing group control (generated by Gang Li). Cells were transfected with 4 mg of each plasmid using Lipofectamine 2000 (Invitrogen) following the manufacturer's instruction. Overexpressing groups were selected with G418 and silencing groups were selected with puromycin (Table 1).

qRT-PCR Analysis

Quantitative PCR (qPCR) was performed to analyze *CBX8*, *WNK2*, and *MMP2* expression in different cell lines. After extraction of total RNA using the Trizol Reagent (Ambion), 500 ng of RNA template was mixed with 5 × PrimeScript RT Master Mix (Bio-Rad) in RNase-Free dH_2O. The mixture was maintained at 37°C for 15 min for reverse transcription, followed by heat inactivation at 85°C for 5 s.

For qPCR, 1 μL of cDNA template was added to a master mix consisting of SYBR Premix EX TaqII (Bio-Rad), forward and reverse primers (Table 2), and ROX reference dye (TaKaRa Bio) to form a 20 μL reaction mixture. Duplicates of each sample were made for each run. The reaction was performed using the 7900HT Fast Real-Time PCR System (Applied Biosystems); the thermal cycling conditions were 30 s at 95°C, followed by 40 cycles of 95°C for 5 s and 60°C for 30 s. The internal loading control was glyceraldehyde-3-phosphate dehydrogenase (GAPDH); the relative *CBX8*, *WNK2*, and *MMP2* expression was calculated with the 2-ΔCt formula, where $\Delta Ct = CtCBX - CtGAPDH$. A list of primers used is included in Table 2.

Western Blot Assay

Protein lysates were generated using radioimmunoprecipitation assay (RIPA) buffer (Cell Signaling Technology) with a protease inhibitor cocktail (Sigma-Aldrich). After incubation for 30 min on ice, the protein lysates were centrifuged at 17,000 rpm for 10 min. Protein concentration was quantified using the bicinchoninic acid method (BCA) (Thermo Fisher Scientific); the optical density (OD) of reaction mixtures was measured at 570 nm, and protein quantities were obtained by referring to the bovine serum albumin standard curve. The samples (20 μg for each lane) paralleled with a protein ladder (10–170 kDa; Thermo Fisher Scientific) were resolved in a 10% SDS-PAGE gel; the proteins were then transferred to polyvinylidene fluoride (PVDF) membranes (GE Healthcare Life Sciences, Vienna, Austria) using a wet electroblotting system (Bio-Rad Laboratories, Pittsburgh, PA, USA). For immunoblotting, the membranes were first blocked with 5% dry fat-free milk for 1 h at room temperature and washed twice with Tris-buffered saline containing 0.1% Tween 20 (TBST; Affymetrix). The membranes were incubated for 2 h at room temperature with different primary antibodies, including anti-CBX8 (Santa Cruz) and anti-GAPDH (Cell Signaling Technology). This was followed by washing twice with TBST and blotting with the corresponding horseradish peroxidase (HRP)-conjugated secondary antibodies (anti-mouse or anti-rabbit; 1:10,000) for 1 h. After the incubation, membranes were washed three times with TBST to remove any non-specific binding of secondary antibody. Chemiluminescent detection of signals was performed with HRP substrates (Merck Millipore), and the signal was visualized in X-ray films.

Wound Healing Assay

Wound healing assay was carried out to determine the cell protrusion and migration ability of tumor cells. U-251 MG, MDA-MB-231, and A549 cells were grown in their respective media and then seeded in 6-well plates and cultured until reaching 100% confluency as a monolayer. A new 20 μL pipette tip was used to make three parallel straight scratches gently and slowly with a marker on the bottom of the dish. The resulting gap distance was considered equivalent to the outer diameter of the end of the tip. Cells were washed with phosphate-buffered saline (PBS) three times and cultured in medium without FBS to inhibit proliferation. Images were acquired using phase contrast and a 10× objective. The wound was measured at 0, 12, 24, 48, and 72 h. Images were analyzed with the online platform WimScratch. Migration was determined by comparing relative gap areas.

Methyl Thiazolyl Tetrazolium (MTT) Assay

The MTT assay was utilized to determine cell viability. Cells were grown in 96-well plates at a density of 5×10^4 cells/well and cultured in medium with or without FBS for 72 h. Then, 20 μL

Table 2. Primers for qRT-PCR

Gene	Forward Primer Chain	Reverse Primer Chain
CBX8	5′-GTGAAATGGAAGGGATG-3′	5′-GTTTTGGGCTTG GGTC-3′
WNK2	5′-GTGCACGATCCTGAAATC-3′	5′-CAGTTTCTTGGG GTCTTCC-3′
MMP2	5′-CTTGACCCATGCATTCTC-3′	5′-CATCCCAATGAC CTCATC-3′
RAC1	5′-GCTTTTGCGGAGATTTTGA-3′	5′-CCCGTGACACTTT CATTCCT-3′
GAPDH	5′-GGTAGGGAGTTCGAGACCAG-3′	5′-TCAACGCAGTTC AGTTAGGC-3′

MTT (5 mg/mL) was added to each well and incubated at 37°C for 4 h. DMSO (150 μL) was added to solubilize the formazan crystals. The plate was then wrapped in foil and placed on an orbital shaker for 15 min. The amount of formazan salt was determined by measuring the OD at 490 nm using a Bio-Rad 680 microplate reader (Bio-Rad Laboratories, Hercules, CA, USA). Cell viability was expressed as the percent OD value of each group relative to that of control cells.

Transwell Migration and Invasion Assays

Cell migration and invasion were assessed using the Millipore 24-well Millicell Chamber with pore size 8 mm (Millipore). For the migration assay, 8,000 cells in DMEM without FBS were added to the upper chamber of the insert. For the invasion assay, 50,000 cells in DMEM without FBS were added to the upper chamber pre-coated with Matrigel (Sigma). DMEM with 20% FBS was placed in the lower chamber. Cells were incubated for 8 h for the migration assay and 20 h for the invasion assay. Then, cells were fixed and stained with 0.1% crystal violet, and non-invading cells were removed with cotton swabs. The number of cells on the lower surface of the chamber membrane was counted under a microscope with a 20× objective in five random fields.

Transfection of siRNAs

Cells were seeded in a 6-well plate at 80%–90% confluence for 24 h without antibiotics. A total of 2,500 ng DNA was diluted with 5.0 μL RNAi. Small interfering RNAs (siRNAs) were from Dharmacon (designated siRNA #1) and Sigma (designated siRNA #2). Cells in all conditions designated as "Control" were transfected with a pool of siRNAs that do not target human genes using Lipofectamine 2000 in 100 μL Opti-MEM Medium without serum, using the manufacturer's protocol. Cells were harvested 24 h after transfection. Pools of at least three siRNAs were used to dilute potential off-target effects.

Tail Vein Injection

8- to 10-week-old NSG mice (Jackson Laboratory) were used. The mice were maintained under specific pathogen-free (SPF) conditions in an animal facility and given a pelleted regular rodent diet and water. Tumor cells were counted and diluted to 5 × 10^6/mL. Prior to injection, mice were weighed and warmed for 10 min in a commercially available warming box to dilate the veins. Then, mice were lightly anesthetized by xylene and ketamine. Approximately 10^6 cells (200 μL) were injected into the tail vein (n = 8). After 6–8 weeks, luciferase imaging was performed to evaluate lung metastasis. Then, mice were euthanized, weighed, and lung tissues were harvested. H&E staining was performed to assess metastasis. All experiments involving mice were performed according to Tianjin Medical University Cancer Institute and Hospital animal guidelines. To generate tumor cell lines stably expressing the luciferase gene, cells were transfected with pGL4.51 plasmids (Promega, WI, USA), followed by G418 selection and confirmation with the presence of luciferin according to manufacture instructions.

ChIP Assay

Formaldehyde (1%) was directly added to cell media. The cross-linking reaction was terminated by adding 0.125 M glycine. Cells were pelleted after centrifugation, followed by preparation of nuclear lysates using Magna ChIP protein G Kit (Millipore, Billerica, MA, USA). Cells were resuspended with 1 mL per 5 × 10^7 cells of swelling buffer. Nuclei were pelleted by microfuge at 2,500 × g for 5 min at 4°C and were resuspended in 1 mL per 1 × 10^8 cells of prepared Nuclei Lysis Solution and incubated on ice for 10 min. Samples were sonicated, then 1 μL of 100 mM phenylmethanesulfonylfluoride (PMSF) was added per 100 μL of blocked Staph A cells. Approximately 1 × 10^7 cells were used for each immunoprecipitation (IP). An immunoglobulin G (IgG) negative control sample was also included. Primary antibodies (1 μg each) were added to each sample and incubated on a rotating platform at 4°C overnight. Secondary antibody (1 μg) was added and incubated for an additional hour at 4°C. The antibody/protein/DNA complexes were eluted by adding 50 μL of IP elution buffer at room temperature, followed by shaking and centrifuging at 14,000 rpm for 3 min at room temperature. Supernatant was removed, then 4 μL of 5 M NaCl (0.2 M NaCl final) was added to each IP sample tube. All samples were incubated at 67°C overnight. Then, 1 μL of 10 mg/mL RNase A was added to each sample and incubated for 30 min at 37°C. ChIP samples were used for PCR reaction.

PCR was performed with primers (Table S1): binding site: 5′-TGGGCAACATGGTGAAA-3′ and 5′-AAGCAATCCTCCCA CCTCA-3′.

RAC1 Activation Assay

RAC1 activity was measured using colorimetric G-LISA assays (Cytoskeleton, Denver, CO, USA) according to the manufacturer's instructions. Briefly, cells were lysed with 1 mg/mL GL36. The lysate was incubated in a RAC1-GTP affinity plate for 30 min on a shaker at 200 rpm. The plate was washed with running buffer, then *RAC1* activity was analyzed on the Molecular Devices SpectraMax 250 at 490 nm.

MMP2 Activation Assay

MMP2 activity was measured using MMP Activity Assay Kit (Fluorometric - Green) (Abcam, USA) according to the manufacturer's

instructions. Briefly, cells were lysed and incubated with 2 mM 4-aminophenylmercuric acetate (APMA) working solution in 96-well plate per assay. Then, 50 μL of MMP Green Substrate working solution was added to the sample and control wells of the assay plate. Fluorescence intensity was assessed with a fluorescence plate reader at Excitation Wavelength (Ex)/Emission Wavelength (Em) = 490/525 nm.

SUPPLEMENTAL INFORMATION

Supplemental Information can be found online at https://doi.org/10.1016/j.omto.2020.09.012.

AUTHOR CONTRIBUTIONS

Y.J., Y.W., and C.Z. conducted the experiment and collected data. Y.J. and M.Y.C. contributed to the conception of the study and wrote the paper.

CONFLICTS OF INTEREST

The authors declare no competing interests.

REFERENCES

1. Borrelli, G.M., Abrão, M.S., Taube, E.T., Darb-Esfahani, S., Köhler, C., Kaufmann, A.M., Chiantera, V., and Mechsner, S. (2015). Immunohistochemical Investigation of Metastasis-Related Chemokines in Deep-Infiltrating Endometriosis and Compromised Pelvic Sentinel Lymph Nodes. Reprod. Sci. *22*, 1632–1642.
2. Yang, A., Fan, H., Zhao, Y., Zha, X., Zhang, H., Hu, Z., and Tu, P. (2016). Huaier aqueous extract inhibits proliferation and metastasis of tuberous sclerosis complex cell models through downregulation of JAK2/STAT3 and MAPK signaling pathways. Oncol. Rep. *36*, 1491–1498.
3. Fidler, I.J., and Kripke, M.L. (2015). The challenge of targeting metastasis. Cancer Metastasis Rev. *34*, 635–641.
4. Cao, H., Xu, E., Liu, H., Wan, L., and Lai, M. (2015). Epithelial-mesenchymal transition in colorectal cancer metastasis: A system review. Pathol. Res. Pract. *211*, 557–569.
5. Friedl, P., and Gilmour, D. (2009). Collective cell migration in morphogenesis, regeneration and cancer. Nat. Rev. Mol. Cell Biol. *10*, 445–457.
6. Sahai, E. (2005). Mechanisms of cancer cell invasion. Curr. Opin. Genet. Dev. *15*, 87–96.
7. Shaverdashvili, K., Wong, P., Ma, J., Zhang, K., Osman, I., and Bedogni, B. (2014). MT1-MMP modulates melanoma cell dissemination and metastasis through activation of *MMP2* and *RAC1*. Pigment Cell Melanoma Res. *27*, 287–296.
8. Wu, D.M., Deng, S.H., Liu, T., Han, R., Zhang, T., and Xu, Y. (2018). TGF-β-mediated exosomal lnc-*MMP2*-2 regulates migration and invasion of lung cancer cells to the vasculature by promoting *MMP2* expression. Cancer Med. *7*, 5118–5129.
9. Mook, O.R., Frederiks, W.M., and Van Noorden, C.J. (2004). The role of gelatinases in colorectal cancer progression and metastasis. Biochim. Biophys. Acta *1705*, 69–89.
10. Pasapera, A.M., Plotnikov, S.V., Fischer, R.S., Case, L.B., Egelhoff, T.T., and Waterman, C.M. (2015). *Rac1*-dependent phosphorylation and focal adhesion recruitment of myosin IIA regulates migration and mechanosensing. Curr. Biol. *25*, 175–186.
11. Pollard, T.D., and Borisy, G.G. (2003). Cellular motility driven by assembly and disassembly of actin filaments. Cell *112*, 453–465.
12. Alvarez, D.E., and Agaisse, H. (2015). A role for the small GTPase *Rac1* in vaccinia actin-based motility. Small GTPases *6*, 119–122.
13. Costa, A.M., Pinto, F., Martinho, O., Oliveira, M.J., Jordan, P., and Reis, R.M. (2015). Silencing of the tumor suppressor gene *WNK2* is associated with upregulation of *MMP2* and JNK in gliomas. Oncotarget *6*, 1422–1434.
14. Moniz, S., Martinho, O., Pinto, F., Sousa, B., Loureiro, C., Oliveira, M.J., Moita, L.F., Honavar, M., Pinheiro, C., Pires, M., et al. (2013). Loss of *WNK2* expression by promoter gene methylation occurs in adult gliomas and triggers *Rac1*-mediated tumour cell invasiveness. Hum. Mol. Genet. *22*, 84–95.
15. Hong, C., Moorefield, K.S., Jun, P., Aldape, K.D., Kharbanda, S., Phillips, H.S., and Costello, J.F. (2007). Epigenome scans and cancer genome sequencing converge on *WNK2*, a kinase-independent suppressor of cell growth. Proc. Natl. Acad. Sci. USA *104*, 10974–10979.
16. Shao, Z., Raible, F., Mollaaghababa, R., Guyon, J.R., Wu, C.T., Bender, W., and Kingston, R.E. (1999). Stabilization of chromatin structure by PRC1, a Polycomb complex. Cell *98*, 37–46.
17. Bernstein, B.E., Mikkelsen, T.S., Xie, X., Kamal, M., Huebert, D.J., Cuff, J., Fry, B., Meissner, A., Wernig, M., Plath, K., et al. (2006). A bivalent chromatin structure marks key developmental genes in embryonic stem cells. Cell *125*, 315–326.
18. Buchwald, G., van der Stoop, P., Weichenrieder, O., Perrakis, A., van Lohuizen, M., and Sixma, T.K. (2006). Structure and E3-ligase activity of the Ring-Ring complex of polycomb proteins Bmi1 and Ring1b. EMBO J. *25*, 2465–2474.
19. van den Boom, V., Maat, H., Geugien, M., Rodríguez López, A., Sotoca, A.M., Jaques, J., Brouwers-Vos, A.Z., Fusetti, F., Groen, R.W., Yuan, H., et al. (2016). Non-canonical PRC1.1 Targets Active Genes Independent of H3K27me3 and Is Essential for Leukemogenesis. Cell Rep. *14*, 332–346.
20. Tavares, L., Dimitrova, E., Oxley, D., Webster, J., Poot, R., Demmers, J., Bezstarosti, K., Taylor, S., Ura, H., Koide, H., et al. (2012). RYBP-PRC1 complexes mediate H2A ubiquitylation at polycomb target sites independently of PRC2 and H3K27me3. Cell *148*, 664–678.
21. Prasad, P., Rönnerblad, M., Arner, E., Itoh, M., Kawaji, H., Lassmann, T., Daub, C.O., Forrest, A.R., Lennartsson, A., and Ekwall, K.; FANTOM consortium (2014). High-throughput transcription profiling identifies putative epigenetic regulators of hematopoiesis. Blood *123*, e46–e57.
22. Lee, S.H., Um, S.J., and Kim, E.J. (2013). *CBX8* suppresses Sirtinol-induced premature senescence in human breast cancer cells via cooperation with SIRT1. Cancer Lett. *335*, 397–403.
23. Tan, J., Jones, M., Koseki, H., Nakayama, M., Muntean, A.G., Maillard, I., and Hess, J.L. (2011). *CBX8*, a polycomb group protein, is essential for MLL-AF9-induced leukemogenesis. Cancer Cell *20*, 563–575.
24. Tang, J., Wang, G., Zhang, M., Li, F.Y., Sang, Y., Wang, B., Hu, K., Wu, Y., Luo, R., Liao, D., et al. (2014). Paradoxical role of *CBX8* in proliferation and metastasis of colorectal cancer. Oncotarget *5*, 10778–10790.
25. Klauke, K., Radulović, V., Broekhuis, M., Weersing, E., Zwart, E., Olthof, S., Ritsema, M., Bruggeman, S., Wu, X., Helin, K., et al. (2013). Polycomb Cbx family members mediate the balance between haematopoietic stem cell self-renewal and differentiation. Nat. Cell Biol. *15*, 353–362.
26. Tang, Y.N., Ding, W.Q., Guo, X.J., Yuan, X.W., Wang, D.M., and Song, J.G. (2015). Epigenetic regulation of Smad2 and Smad3 by profilin-2 promotes lung cancer growth and metastasis. Nat. Commun. *6*, 8230.
27. Zingg, D., Debbache, J., Schaefer, S.M., Tuncer, E., Frommel, S.C., Cheng, P., Arenas-Ramirez, N., Haeusel, J., Zhang, Y., Bonalli, M., et al. (2015). The epigenetic modifier EZH2 controls melanoma growth and metastasis through silencing of distinct tumour suppressors. Nat. Commun. *6*, 6051.
28. Li, G., Warden, C., Zou, Z., Neman, J., Krueger, J.S., Jain, A., Jandial, R., and Chen, M. (2013). Altered expression of polycomb group genes in glioblastoma multiforme. PLoS ONE *8*, e80970.
29. Gao, S.B., Sun, S.L., Zheng, Q.L., Zhang, L., Zhu, Y., Jin, G.H., and Xue, L.X. (2015). Genetic alteration and misexpression of Polycomb group genes in hepatocellular carcinoma. Am. J. Cancer Res. *5*, 2969–2979.
30. Yuan, G.J., Chen, X., Lu, J., Feng, Z.H., Chen, S.L., Chen, R.X., Wei, W.S., Zhou, F.J., and Xie, D. (2017). Chromobox homolog 8 is a predictor of muscle invasive bladder cancer and promotes cell proliferation by repressing the p53 pathway. Cancer Sci. *108*, 2166–2175.
31. Wang, G., Tang, J., Zhan, W., Zhang, R., Zhang, M., Liao, D., Wang, X., Wu, Y., and Kang, T. (2017). *CBX8* Suppresses Tumor Metastasis via Repressing Snail in Esophageal Squamous Cell Carcinoma. Theranostics *7*, 3478–3488.

OMTO, Volume *19*

Supplemental Information

Upregulated *CBX8* Promotes Cancer Metastasis via the *WNK2*/*MMP2* Pathway

Yongsheng Jia, Yujun Wang, Cuicui Zhang, and Mike Yue Chen

Table S1: Primers for CHIP

primers	Forward primer chain	Reverse primer chain
Primer 1	CACCTCACACCTGTTAAGATC	CACTCACACCACACTGCAC
Primer 2	GCCAAGATATGGAAACAACC	GTACTCCACTGTGCAAAC
Primer 3	CCAGGCACAGAAAGACTAC	CAACTTCCATTCTGCTCTCTGC
Primer 4	GTGTTAAGACAGCAGATCTC	CCTAGGGCTGTGTGATGGCGTC
Primer 5	CACGGGCCTGCACGCACTGTG	CATTCTGTGTTGGTACTCAC
Primer 6	TGGGCAACATGGTGAAA	AAGCAATCCTCCCACCTCA
Primer 7	CAGAGGTTGCAGTGAGGCGAG	GATGGTCTGGCTCTGTCAC
Primer 8	GTCCCTGTGAGCCTGGTGGAG	CCTGTGGGAGGAATCCAGAACC
Primer 9	GAGCTCCTGCACAAGGCACTG G	AGGAGCCTGAAGCCTTCAGACC
Primer 10	GCCACAGCTGATGGTCACACG	GAGCCACCAGCTTCGCAACTCC
Primer 11	GAGAAGACCAGCACGCTGG	GTACCAGGGTACTCCAGTCC

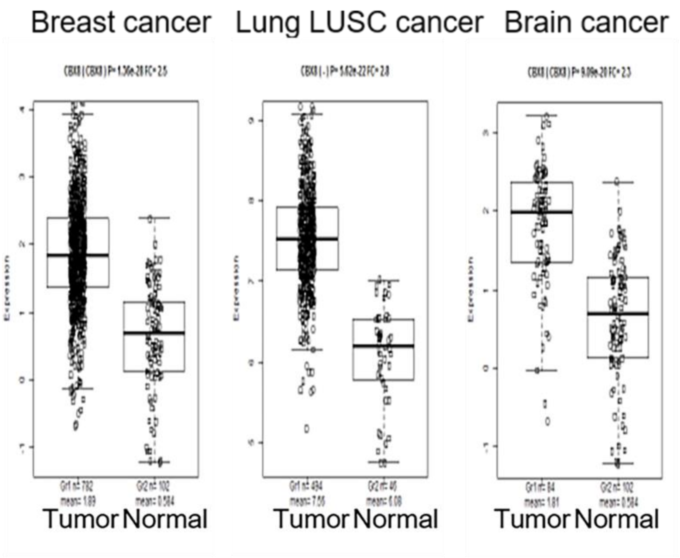

Figure S1. *CBX8* expression is analyzed in breast cancer, lung LUSC cancer, and brain cancer tissues compared to normal tissues. *CBX8* shows significant upregulation from our in-house ANOVA difference gene expression analysis using TCGA GDAC provisional patient cohorts for brain cancer (GBM with 525 tumor vs 10 normal samples), breast cancer (BRCA, with 1100 tumor vs 112 normal samples), and lung cancer (LUAD, with 517 tumor vs 59 normal samples).

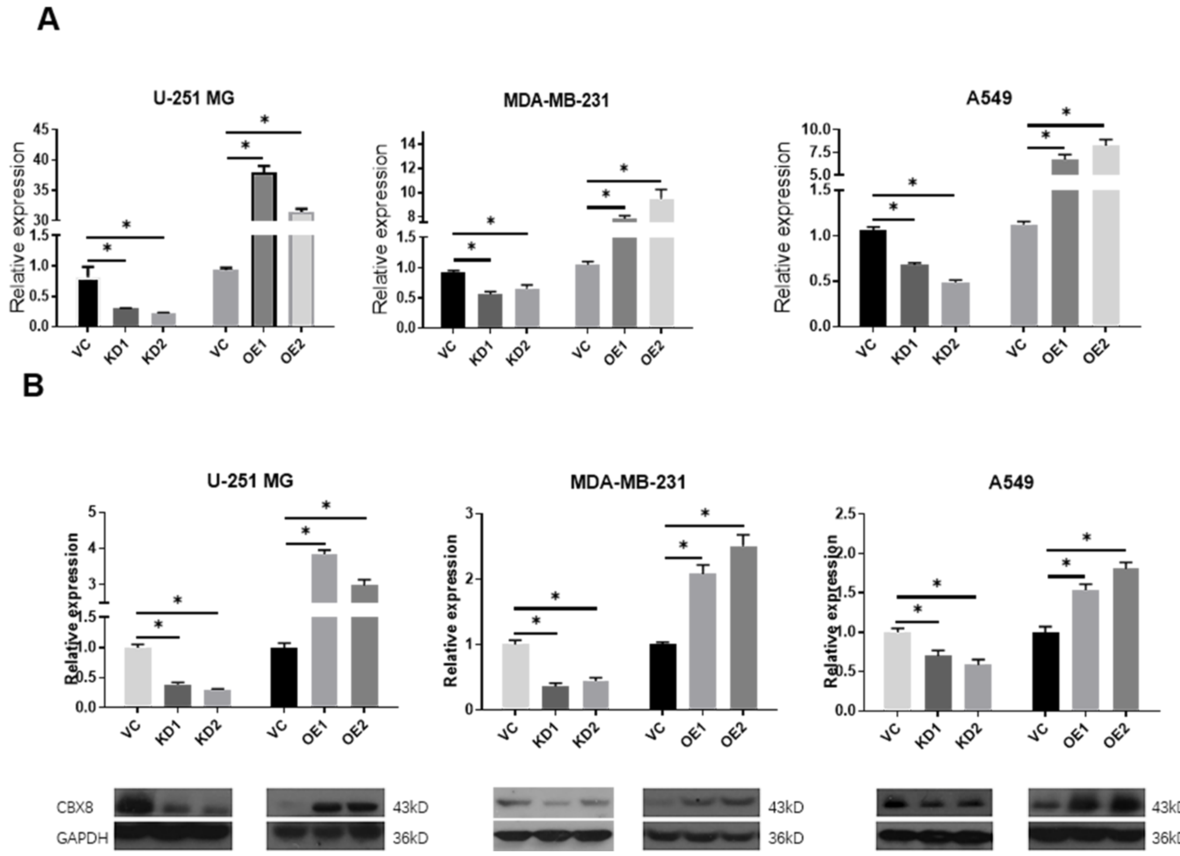

Figure s2: *CBX8* modulated in cancer cells and evaluated by qRT-PCR (A) and western blot (B). “*” indicated p<0.05.

MDA-MB-231

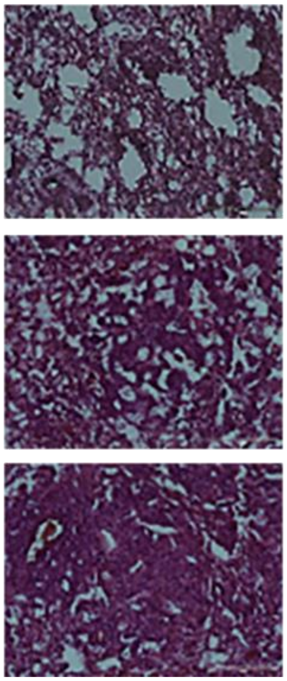

Figure S3. H&E staining of lung tissues from mice injected with CBX8-modulated MDA-MB-231 cells as well as corresponding vector controls.

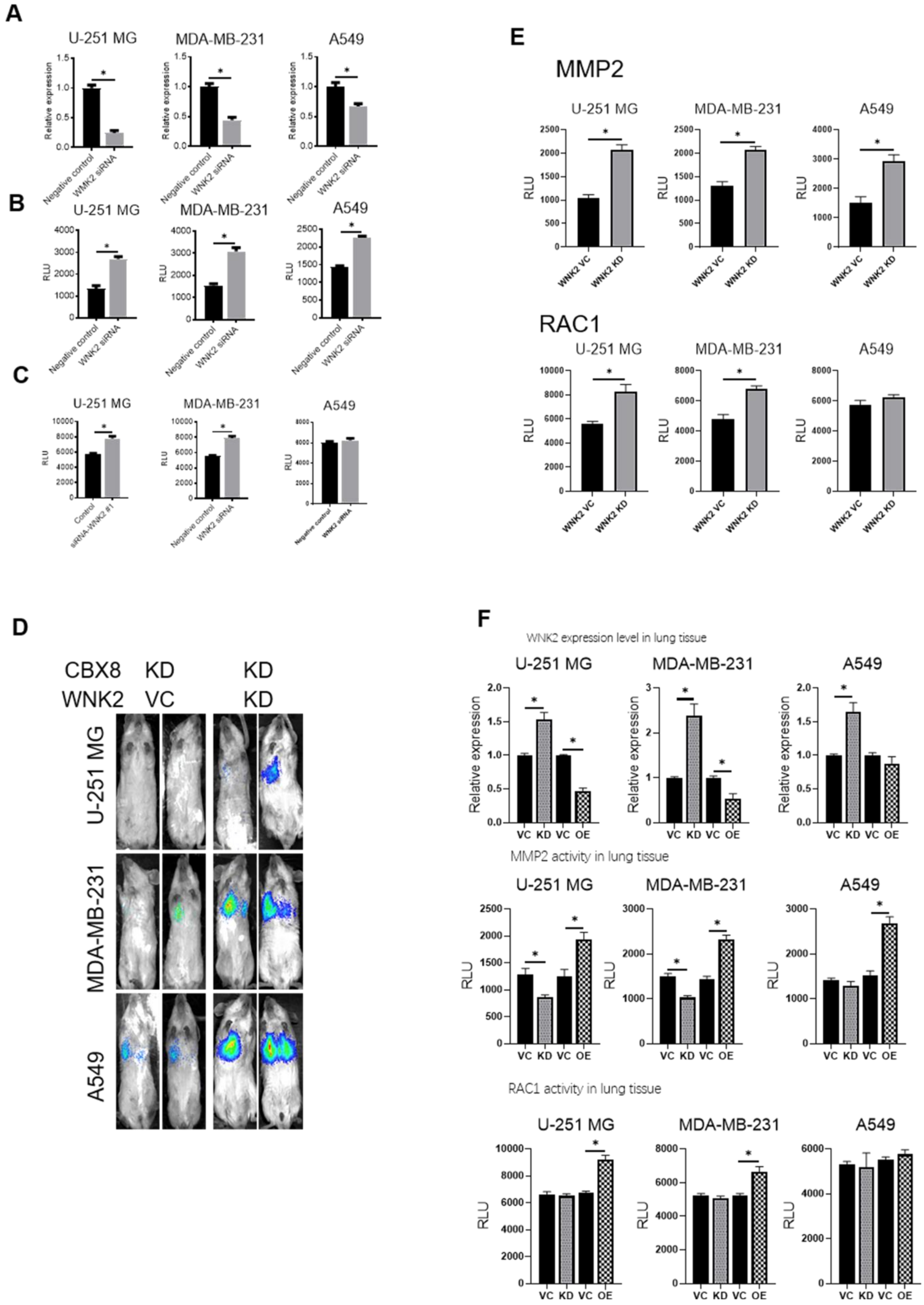

A
U-251 MG
MDA-MB-231
A549
Relative expression
Negative control
WMK2 siRNA
WNK2 siRNA
B
RLU
C
Control
siRNA-WNK2 #1
D
CBX8
WNK2
KD
VC
KD
KD
E
MMP2
WNK2 VC
WNK2 KD
RAC1
F
WNK2 expression level in lung tissue
VC KD VC OE
MMP2 activity in lung tissue
RAC1 activity in lung tissue

Figure S4. Knockdown of *WNK2* increases the RAC1 and MMP2 activity in tumor cells A. Transient transfection of SiRNA targeting *WNK2* suppress its expression. B and C. Knockdown of *WNK2* enhances the MMP2 and RAC1 activity in U-251 MG, MDA-MB-231, and A549 tumor cells. WNK2 promoter activity increased in U-251MG, MDA-MB-231 and A549 cells after knockdown of CBX8(E). The expression of WNK2, MMP2 and RAC1 are assessed in the tumor tissues of the in vivo mouse model(F). "*" indicated $p<0.05$.
